# Supplementary material for: Mapping and quantifying travel time to define health facility catchment areas in Blantyre city in Malawi
Source: Commun Med (Lond). 2025 Jun 11;5:227. doi: 10.1038/s43856-025-00845-3 (PMC12159157; doi:10.1038/s43856-025-00845-3)

Supplementary information

Supplementary tables

Supplementary table 1: Observed travel speed based on OSM highway tags

Highway tag	Mode of transportation	Average travel speed (m/s)	Standard Error	Average travel speed (km/hr)	Applicable to
Residential	Bicycle	2.89	0.25	10.40	Residential & service
Residential	Car	3.85	0.90	14.86	Residential & service
Residential	Motorcycle	5.06	0.55	18.22	Residential & service
Residential	Walking	1.41	0.08	5.07	Residential & service
Tertiary	Bicycle	4.82	0.41	17.35	Primary, secondary & tertiary
Tertiary	Car	8.19	0.31	29.48	Primary, secondary & tertiary
Tertiary	Motorcycle	9.14	0.38	32.90	Primary, secondary & tertiary
Tertiary	Walking	1.47	0.25	5.29	Primary, secondary & tertiary
Track	Walking	1.34	0.04	4.82	Applicable to footway, path, track, trunk & cycleway
Unclassified	Unclassified	1.39	0.08	5	Applicable to all unclassified roads

Supplementary table 2: Analysis of variance table from the analysis to compare the travel speed between different models of transportation. The Analysis of Variance for the observations on travel speed computed when means of transportation, road surface and location is taken into account

	DF	Sum Sq	Mean Sq	F-Value	Pr(>F)
Transportation	3	4999	1666.5	626.953	<2e-16 ***
Surface	2	774	387.1	145.644	<2e-16 ***
Location	6	318	53.1	19.965	<2e-16 ***
Transportation: Surface	3	508	169.5	63.764	<2e-16 ***
Transportation: Location	12	298	24.9	9.355	<2e-16 ***
Surface: Location	4	12	3.1	1.160	0.3274
Transportation: Surface-Location	5	31	6.2	2.343	0.0402 *
Residuals	595	1582	2.7		

Supplementary table 3: Population estimate within each public health facility catchment using WorldPop data. See main text for how population was corrected.

ID	Name	Ownership	District	Worldpop estimate	Corrected population
1	Bangwe Clinic	Public	Blantyre	117,187	101,401
2	Makhetha Dispensary	Public	Blantyre	85,818	74,257
3	Limbe Health Center	Public	Blantyre	33,029	28,580
4	Ndirande Health Centre	Public	Blantyre	64,481	55,795
5	Gate Way Clinic	Public	Blantyre	42,798	37,033
6	Chilomoni Health Centre	Public	Blantyre	48,543	42,003
7	Mbatani Health Centre	Public	Blantyre	74,388	64,367
8	Chirimba Health Centre	Public	Blantyre	114,833	99,363
9	South Lunzu Health Centre	Public	Blantyre	118,404	102,453
10	Chichiri Prison Clinic	Public	Blantyre	24,213	20,951
11	Kanjedza Police Clinic	Public	Blantyre	26,785	23,177
12	Zingwangwa Urban Health Centre	Public	Blantyre	129,883	112,386
13	Macro Blantyre	Public	Blantyre	25,907	22,417
14	Sanjika Clinic	Public	Blantyre	11,274	9,755

Supplementary table 4: Population estimate within each private health facility catchment, using from WorldPop and corrected to match census data. See main text for how population was corrected.

ID	Name	Ownership	District	Worldpop estimate	Corrected population
1	SOS Childrens Village Blantyre	Private	Blantyre	131,136	113,471
2	Malmed Private Clinic	Private	Blantyre	23,584	20,407
3	Blantye adventist Hospital	Private	Blantyre	127,210	110,074
4	Banja lamtsogolo Bangwe Clinic	Private	Blantyre	231,530	200,340
5	Lungu private Clinic	Private	Blantyre	14,297	12,371
6	Madinah social services clinic	Private	Blantyre	30,508	26,398
7	Blantyre Dream Project	Private	Blantyre	12,528	10,840
8	Mwaiwathu Clinic	Private	Blantyre	5,803	5,021
9	Polytechnic Clinic	Private	Blantyre	7,400	6,403
10	soche SDA Dispensary	Private	Blantyre	150,960	130,624
11	Shifa	Private	Blantyre	1,957	1,693
12	Malabada Health Centre	Private	Blantyre	40,385	34,944
13	SOBO Clinic	Private	Blantyre	1,589	1,375
14	Nyambadwe Private Hospital	Private	Blantyre	46,136	39,921
15	Mwayiwathu Private Hospital	Private	Blantyre	2,460	2,129
16	Blantyre Waterboard Clinic	Private	Blantyre	2,507	2,169
17	Chichiri Escom Clinic	Private	Blantyre	6,366	5,508
18	Mapeto DWS Clinic	Private	Blantyre	10,907	9,438
19	Lafarge Cement Clinic	Private	Blantyre	5,604	4,849
20	Dr. S. kampondeni	Private	Blantyre	89,109	77,105
21	Blantyre city assembly Clinic	Private	Blantyre	12,079	10,452
22	Malamulo Day Clinic. Amina	Private	Blantyre	65,697	56,847
23	Kanjedza Masm Medi Clinic	Private	Blantyre	32,721	28,313
24	Mtengo Umodzi Private Hospital	Private	Blantyre	126,799	109,718

Supplementary figures

Supplementary figure 1: Workflow of the methods employed in the study

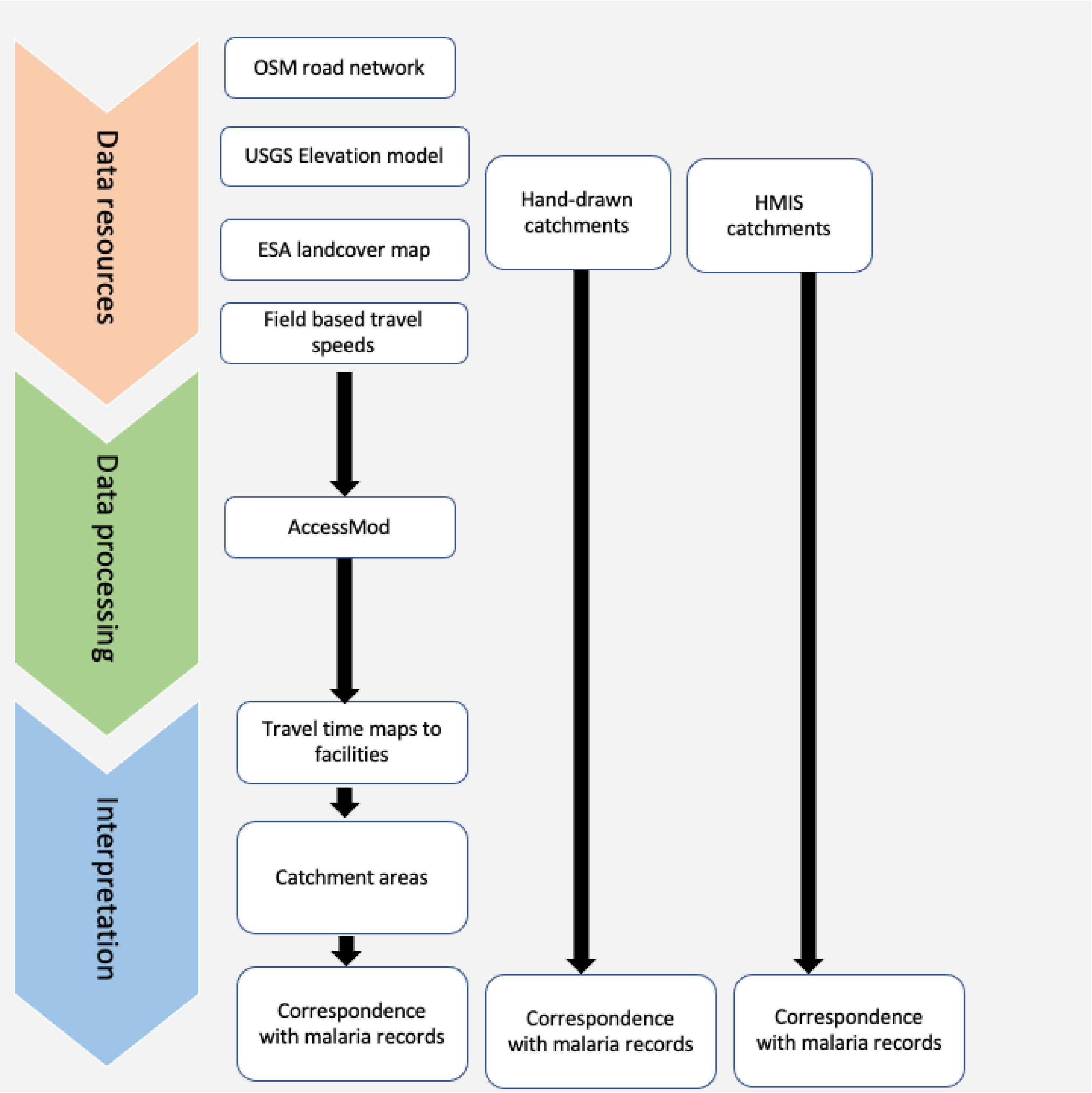

Supplementary figure 2: An example of health facility catchment area as defined by Health Surveillance Assistants.

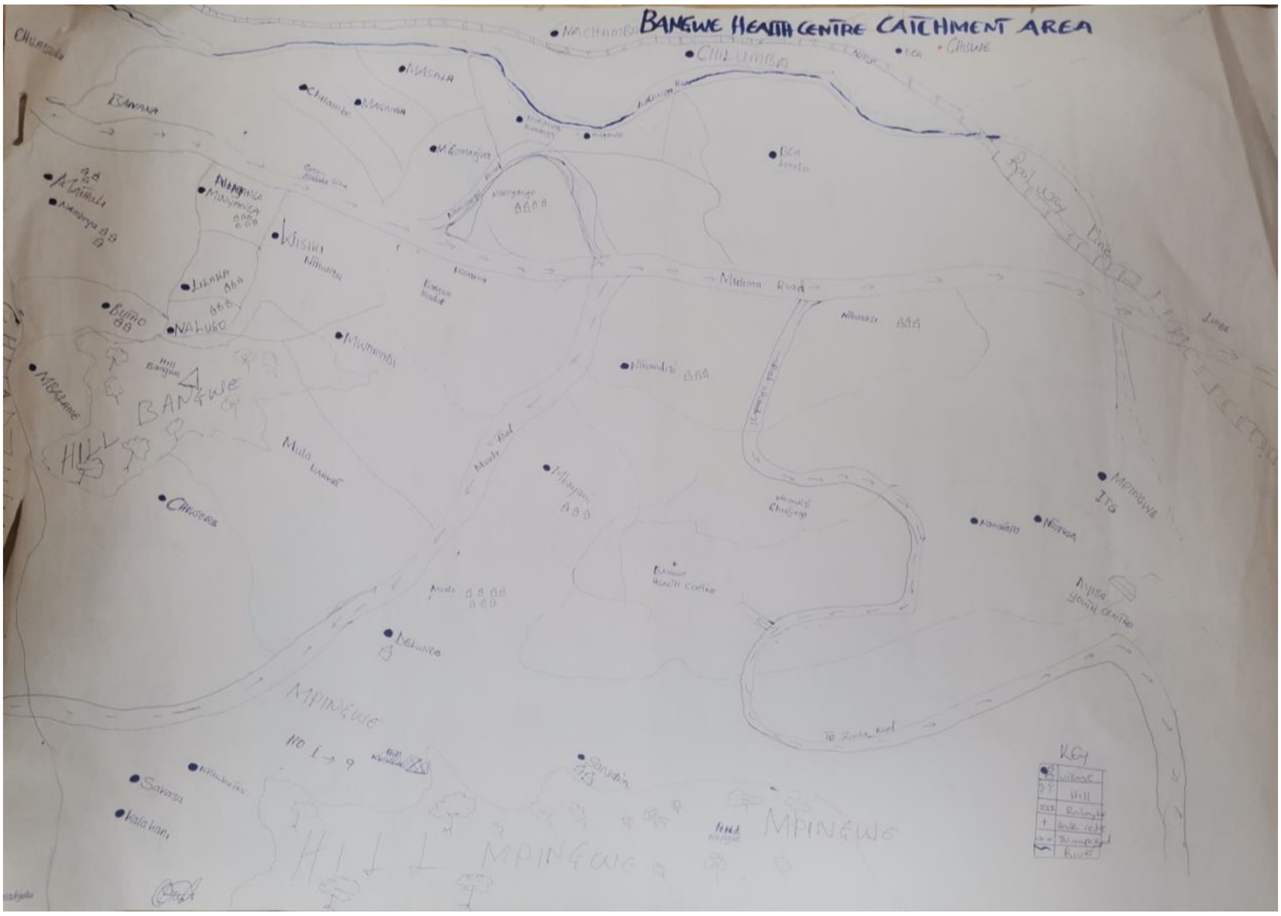

Supplementary figure 3: Sankey diagram for patients accessing health facilities that were considered for validation

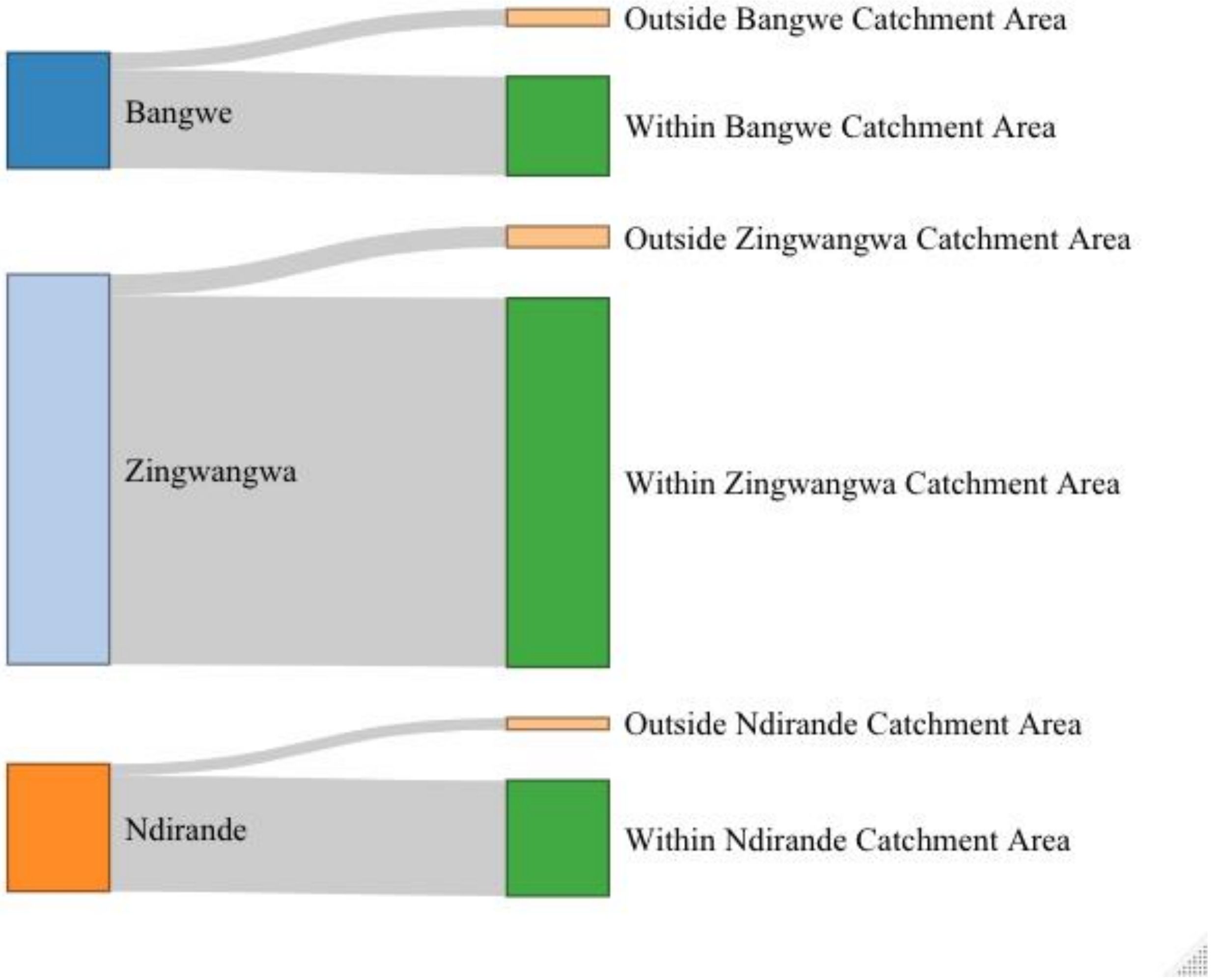

Supplementary figure 4: Catchment areas generated using other travel scenarios observed in the study.

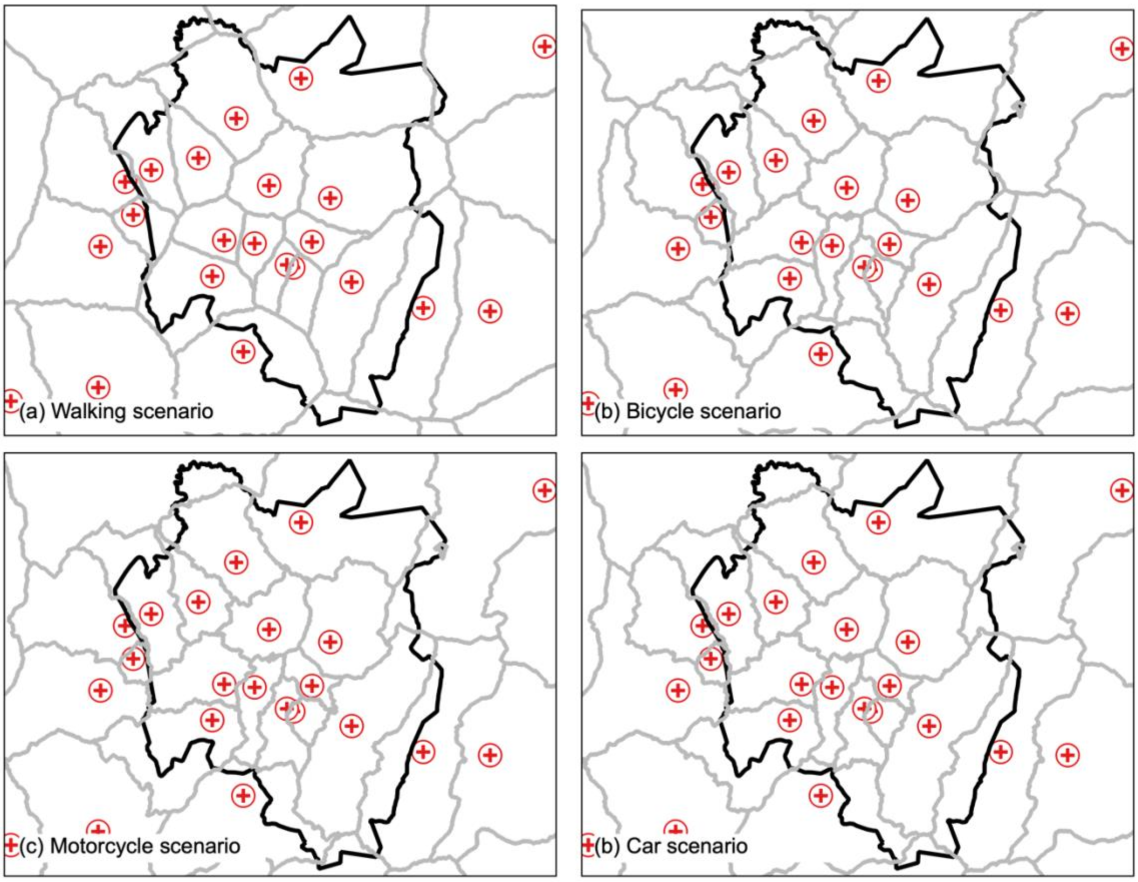

Supplementary figure 5: a hand-drawn catchment for Ndirande Health Centre. **b** digitized map of the same facility overlaid on the boundaries of the modelled catchment area of the same facility.

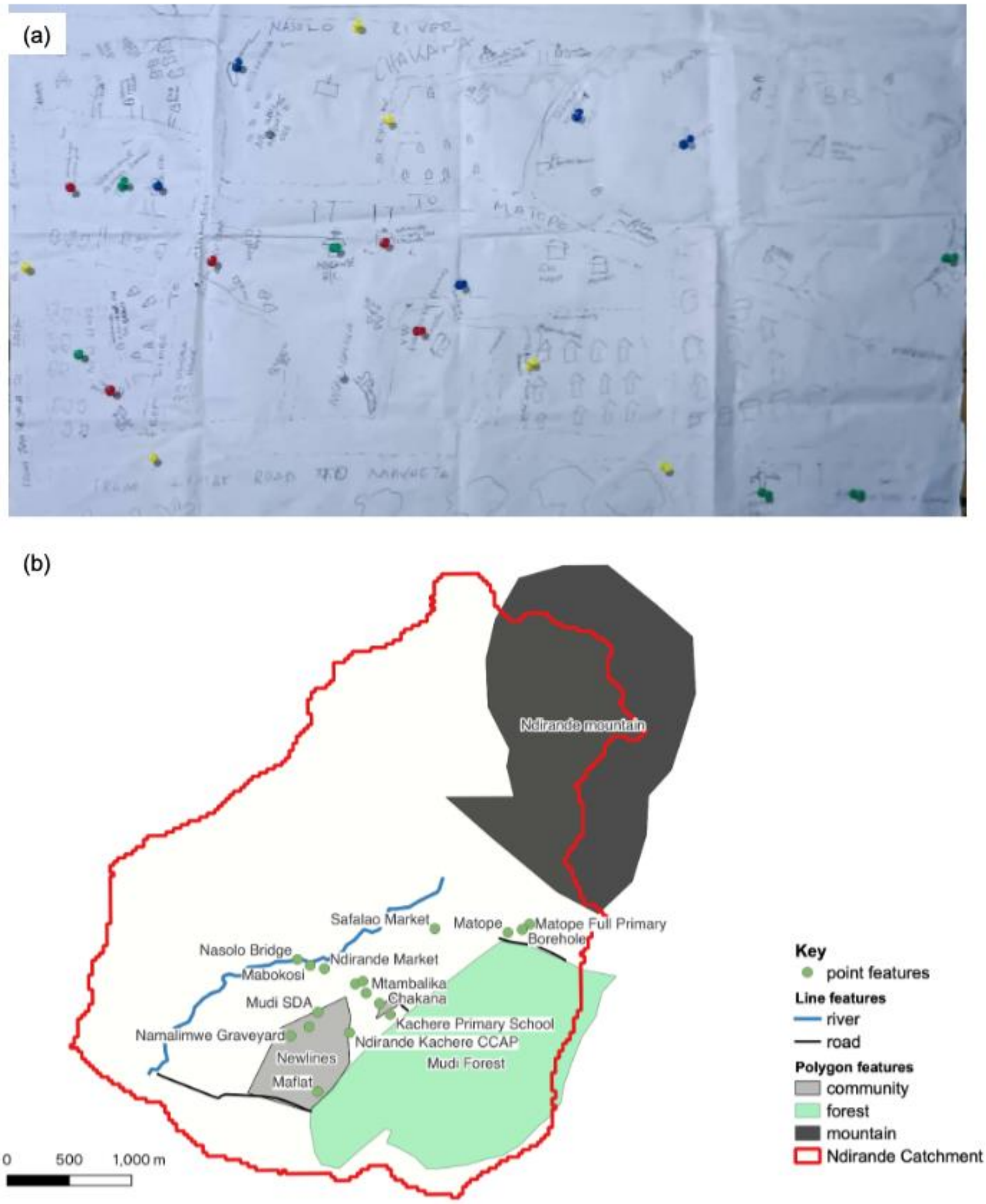

Supplement: Supplementary file 2 — Supplementary file [file 43856_2025_845_MOESM2_ESM.pdf]
